# Aberrant Intrinsic Connectivity of Hippocampus and Amygdala Overlap in the Fronto-Insular and Dorsomedial-Prefrontal Cortex in Major Depressive Disorder

**DOI:** 10.3389/fnhum.2013.00639

**Published:** 2013-10-01

**Authors:** Masoud Tahmasian, David C. Knight, Andrei Manoliu, Dirk Schwerthöffer, Martin Scherr, Chun Meng, Junming Shao, Henning Peters, Anselm Doll, Habibolah Khazaie, Alexander Drzezga, Josef Bäuml, Claus Zimmer, Hans Förstl, Afra M. Wohlschläger, Valentin Riedl, Christian Sorg

**Affiliations:** ^1^Department of Neuroradiology, Klinikum rechts der Isar, Technische Universität München, Munich, Germany; ^2^Department of Nuclear Medicine, Klinikum rechts der Isar, Technische Universität München, Munich, Germany; ^3^TUM-Neuroimaging Center (TUM-NIC), Klinikum rechts der Isar, Technische Universität München, Munich, Germany; ^4^Sleep Disorders Research Center, Department of Psychiatry, Kermanshah University of Medical Sciences (KUMS), Kermanshah, Iran; ^5^Department of Psychology, University of Alabama at Birmingham (UAB), Birmingham, AL, USA; ^6^Department of Psychiatry, Klinikum rechts der Isar, Technische Universität München, Munich, Germany; ^7^Christian Doppler Klinik, Paracelsus Medical University, Salzburg, Austria; ^8^Graduate School of Systemic Neurosciences, Ludwig Maximilians University, Munich, Germany; ^9^Department of Nuclear Medicine, University Hospital of Cologne, Cologne, Germany; ^10^Department of Neurology, Klinikum rechts der Isar, Technische Universität München, Munich, Germany

**Keywords:** major depressive disorder, hippocampus, amygdala, dorsomedial-prefrontal cortex, fronto-insular operculum, resting-state fMRI, intrinsic functional connectivity

## Abstract

Neuroimaging studies of major depressive disorder (MDD) have consistently observed functional and structural changes of the hippocampus (HP) and amygdale (AY). Thus, these brain regions appear to be critical elements of the pathophysiology of MDD. The HP and AY directly interact and show broad and overlapping intrinsic functional connectivity (iFC) to other brain regions. Therefore, we hypothesized the HP and AY would show a corresponding pattern of aberrant intrinsic connectivity in MDD. Resting-state functional MRI was acquired from 21 patients with MDD and 20 healthy controls. ß-Maps of region-of-interest-based FC for bilateral body of the HP and basolateral AY were used as surrogates for iFC of the HP and AY. Analysis of variance was used to compare ß-maps between MDD and healthy control groups, and included covariates for age and gender as well as gray matter volume of the HP and AY. The HP and AY of MDD patient’s showed an overlapping pattern of reduced FC to the dorsomedial-prefrontal cortex and fronto-insular operculum. Both of these regions are known to regulate the interactions among intrinsic networks (i.e., default mode, central executive, and salience networks) that are disrupted in MDD. These results provide the first evidence of overlapping aberrant HP and AY intrinsic connectivity in MDD. Our findings suggest that aberrant HP and AY connectivity may interact with dysfunctional intrinsic network activity in MDD.

## Introduction

Major depressive disorder (MDD) is one of the most common psychiatric disorders with a lifetime prevalence of about 16% (Kessler et al., 2003). It is characterized by episodes of prolonged depressed mood, reduced energy, impaired cognition, vegetative symptoms, and suicidal tendencies with suicide rates of nearly 4% (American Psychiatric Association, 2000). Recent work indicates that MDD is associated with dysfunction of emotion/emotion regulation-related circuitry that includes the hippocampus (HP) and amygdale (AY) among other brain regions (Drevets et al., 2008). The strong consistency of structural and functional HP and AY changes observed across a number of studies highlights the prominent role of these two brain regions in the pathophysiology of MDD (Rigucci et al., 2010; Kempton et al., 2011; MacQueen and Frodl, 2011; Murray et al., 2011). The current study assessed HP and AY pathophysiology in MDD with special focus on the HP’s and AY’s intrinsic functional connectivity (iFC), an index of the synchrony between ongoing HP/AY activity and the ongoing activity of other brain areas.

The HP and AY are located adjacent to one another within the medial temporal lobes, and interact extensively with one another as well as within widely distributed cortical-subcortical circuits (Squire et al., 2004; LeDoux, 2007; Aggleton, 2012). Together, they contribute to several cognitive-behavioral functions, particularly to emotional memory (LaBar and Cabeza, 2006), which is impaired in MDD (e.g., increased negative memory bias in MDD) (Hamilton and Gotlib, 2008).

Resting-state functional MRI (rs-fMRI), which is commonly used to analyze intrinsic connectivity (Fox and Raichle, 2007), has revealed specific iFC patterns for both HP and AY in healthy subjects (Kahn et al., 2008; Etkin et al., 2009; Roy et al., 2009). These HP and AY iFC patterns are altered in patients with MDD (Cao et al., 2012; Tang et al., 2012). For example, resting-state activity within the posterior HP is positively correlated with areas of the “task-negative” network (Fox et al., 2005; Fox and Raichle, 2007) and negatively correlated with resting-state activity within a widely distributed set of brain regions involved in a large range of tasks [i.e., “task-positive” network including the so-called salience and central executive network (Fox et al., 2005; Dosenbach et al., 2007; Seeley et al., 2007)]. The default mode, salience, and central executive networks are intrinsic networks of synchronized, ongoing activity that are associated with specific symptoms (e.g., rumination) when disrupted in MDD (Greicius et al., 2007; Sheline et al., 2009, 2010; Hamilton et al., 2012, 2013). Recent research has demonstrated aberrant hippocampal iFC with areas of the task-negative network in MDD (Cao et al., 2012). Prior research has also shown that resting-state activity within the basolateral AY is positively correlated with the activity of primary areas (such as those of the visual or sensorimotor system), the medial prefrontal cortex (PFC), and of the medial temporal lobe, but negatively correlated with areas of the salience network [i.e., the dorsomedial PFC and fronto-insular operculum (FIO)] (Etkin et al., 2009; Roy et al., 2009; Veer et al., 2010). This last finding is also supported by findings in rats (i.e., negative correlations between ongoing amygdala activity and activity in a frontolimbic circuit), suggesting the robustness of the AY intrinsic connectivity pattern across species (Liang et al., 2012). Recent research has also observed aberrant amygdala iFC with the PFC in first-episode MDD (Tang et al., 2012). Due to the intimate interaction between the HP and AY as well as the partly overlapping cortical iFC patterns of the HP and AY, the question arises as to whether aberrant HP and AY iFC to the cortex might be linked in MDD. This question is important for a better understanding of the pathophysiology of MDD, because a correspondence between aberrant HP and AY iFC may link the medial temporal lobe changes of MDD to the aberrant cortical intrinsic networks in MDD (Menon, 2011; Hamilton et al., 2013). Based on the studies described above, we hypothesized that MDD patients would show a spatially overlapping pattern of aberrant HP and AY intrinsic connectivity with the cortex.

To address this hypothesis, we assessed 21 patients with MDD and 20 age, gender, and education matched healthy controls using blood oxygenation level dependent (BOLD) rs-fMRI signal fluctuations. The primary outcome measure for this study was ß-maps reflecting region-of-interest (ROI) based positive and negative BOLD correlations as a surrogate of iFC. ROIs were positioned bilaterally within the basolateral AY and body of HP. These regions have extensive cortical iFC covering intrinsic networks such as default mode, salience, and central executive network (Kahn et al., 2008; Etkin et al., 2009; Roy et al., 2009). Since we were not interested in lateralized effects of left and right AY/HP, iFC, group comparisons for bilateral AY and HP connectivity were framed by an analysis of variance (ANOVA) approach with factors group and brain side, in which the main effect of group was the effect of interest. Prior research has observed structural changes in the HP and AY of MDD patients (Kempton et al., 2011). Therefore, covariates for HP and AY gray matter (GM) volume were included in the analysis to ensure our findings were independent of differences in HP and AY structure between MDD and healthy control groups.

## Materials and Methods

### Participants

Twenty-one patients with recurrent MDD [female/male: 11/10; mean age: 51.0 (SD 15.0)] and 20 healthy controls [female/male: 11/9; mean age: 49.6 (SD 13.9)] participated in this study (Table 1). After receiving the approval from the medical ethical board of Technische Universität München (TUM), all participants provided informed consent in accordance with the Human Research Committee guidelines of the Klinikum Rechts der Isar, TUM. All patients were inpatients recruited from the Department of Psychiatry, TUM. Controls were recruited from the larger community by word-of-mouth advertising and group-matched by age, gender, and education to MDD patients. Participants’ examination included medical history, psychiatric interview, psychometric assessment, and blood tests for patients. Psychiatric diagnoses were based on DSM-IV (American Psychiatric Association, 2000). The Structured Clinical Interview for DSM-IV (CID-I; First et al., 1996) was used to assess the presence of psychiatric diagnoses. The severity of clinical symptoms was measured with the Hamilton Rating Scale for Depression (HAM-D) (Hamilton, 1960) as well as the Beck Depression Inventory (Beck et al., 1961). The global level of social, occupational, and psychological functioning was measured with the Global Assessment of Functioning (GAF) scale (Endicott et al., 1976). Psychiatrists Dirk Schwerthöffer and Martin Scherr performed clinical-psychometric assessment. They have been professionally trained for SCID interviews with inter-rater reliability for diagnosis of more than 95%.

**Table 1:** **Demographic and clinical data**.

	Patients with MDD (*n* = 21)	Healthy controls (*n* = 20)	*p*-Value
Gender (female)	11	11	>0.05
Age (years)	51.0 (15.0)	49.6 (13.9)	>0.05
Education (years)	14.3 (2.1)	15.2 (2.0)	>0.05
BDI	25.3 (7.1)	0.3 (1.2)	<0.01
HDRS	23.8 (7.9)	0.5 (1.0)	<0.01
GAF	46.7 (11.3)	100 (0)	<0.01
Duration of MDD (years)	14.7 (10.9)	–	–
Age of onset	35.0 (13.6)	–	–
Number episodes	5.0 (2.5)	–	–
Duration of current episode (weeks)	16.2 (6.6)		–
Anti-depressive medication (mono-/bi-/triple therapy)	5/10/5	0/0/0	–
Psychiatric co-morbidity (axis I)	8	0	–

MDD, Major depression disorder; BDI, Beck depression inventory; HDRS, Hamilton depression rating scale; GAF, Global Assessment of Functioning Scale; group comparisons: χ^2^ (gender), two-sample *t*-test (age, education, BDI, HDRS, GAF).

Major depressive disorder was the primary diagnosis for all patients. All patients met criteria for a current major depressive episode with an average episode length of 16.2 weeks (range 8–31 weeks). The average age of MDD onset was 35 years, and all patients experienced their first MDD episode before 50 years of age. The average total duration of MDD was 14.7 years and on average, patients had experienced five depressive episodes. Four patients had a positive family history of MDD. Thirteen MDD patients had co-morbid diagnoses: six generalized anxiety disorder, two somatization disorder, and five avoidant or dependent personality disorders. Patients with psychotic symptoms, schizophrenia, bipolar disorder, and substance abuse were excluded from this study. Additional exclusion criteria were age below 18 or above 70 years, pregnancy, neurological or internal systemic diseases, and general contraindications for MRI. One patient was free of any psychotropic medication during MRI assessment. Five patients were treated by antidepressant mono-therapy (two cases: citalopram 30 mg/day (mean dose), two cases: sertraline 200 mg/day, one case: mirtazapine 30 mg/day), 10 patients by dual-therapy (four cases: citalopram 37.5 mg/day; mirtazapine 30 mg/day, one case: citalopram 40 mg/day; venlafaxine 225 mg/day, one case: citalopram 30 mg/day; quetiapine 200 mg/day, one case: sertraline 200 mg/day; mirtazapine 30 mg/day, three cases: venlafaxine 225 mg/day; mirtazapine 30 mg/day), and five patients by tri-therapy (two cases: citalopram 30 mg/day; venlafaxine 187.5 mg/day; amisulprid 200 mg/day, two cases: citalopram 30 mg/day; mirtazapine 30 mg/day; quetiapine 200 mg/day, one case: venlafaxine 22 mg/day; mirtazapine 30 mg/day; quetiapine 200 mg/day). All healthy controls were free of any current or past neurological or psychiatric disorder or psychotropic medication.

All participants underwent a 5-min structural MRI and 10-min rs-fMRI with the instruction to keep their eyes closed and not to fall asleep. Subjects were questioned via intercom immediately after the rs-fMRI scan to verify they remained awake during the scan. Before and after scanning, a medical examination of patients validated their condition was stable and determined whether they experienced any odd sensations during the scanning. No unusual sensations were reported, and no patient dropped out during the scanning session.

### MRI data acquisition

MRI was performed on a 3-T whole body MR scanner (Achieva, Philips, Netherlands) using an eight-channel phased-array head coil. fMRI data were collected using a gradient echo EPI sequence (TE = 35 ms, TR = 2000 ms, flip angle = 82°, FoV = 220 mm × 220 mm, matrix = 80 × 80, 32 slices, slice thickness = 4 mm, and 0 mm interslice gap; 10 min of scanning resulting in 300 volumes). T1-weighted structural data were obtained using a magnetization-prepared rapid acquisition gradient echo sequence (TE = 4 ms, TR = 9 ms, TI = 100 ms, flip angle = 5°, FoV = 240 mm × 240 mm, matrix = 240 × 240, 170 slices, voxel size = 1 mm × 1 mm × 1 mm).

### rs-fMRI data analysis

The first three functional images of each subject’s dataset were discarded due to magnetization effects. The remaining rs-fMRI data were preprocessed in SPM8 (Wellcome Department of Cognitive Neurology, London) including head motion correction, spatial normalization into the stereotactic space of the Montreal Neurological Institute (MNI) with isotropic voxels of 3 mm × 3 mm × 3 mm, and spatial smoothing with a 6 mm × 6 mm × 6 mm Gaussian kernel to reduce spatial noise. To ensure data quality, particularly concerning motion-induced artifacts, temporal signal-to-noise ratio (tSNR) and point-to-point head motion were estimated for each subject (Murphy et al., 2007; Van Dijk et al., 2012). Excessive head motion (cumulative motion translation or rotation >3 mm or 3° and mean point-to-point translation or rotation >0.15 mm or 0.1°) was applied as exclusion criterion. Point-to-point motion was defined as the absolute displacement of each brain volume compared to its previous volume. None of the participants had to be excluded. Two-sample *t*-tests yielded no significant differences between groups in mean point-to-point translation or rotation in any direction (*p* > 0.10) or in tSNR (*p* > 0.50). Further control for head motion effects was carried out at subject-level iFC analysis.

#### Subject-level iFC analysis

**Figure 1:**
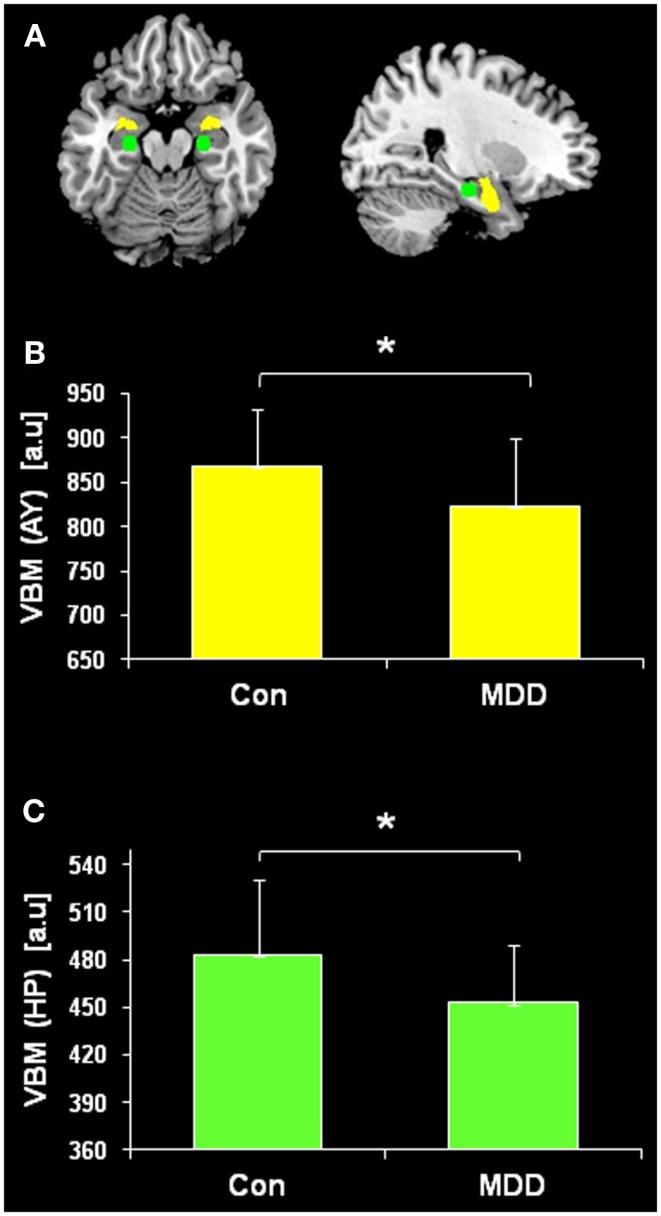
**Regional hippocampus (HP) and amygdala (AY) volumes**. Seeds for the iFC analysis were positioned in the basolateral AY (yellow) and the body of HP (green). Regional voxel-based morphometry (VBM) volumes from the basolateral AY and body of HP were averaged across hemispheres and compared between patients with major depressive disorder (MDD) and healthy controls (Con) using two-sample *t*-tests (*p* < 0.05). HP (*p* = 0.011) and AY (*p* = 0.019) volumes were reduced in MDD patients.

Seeds for the iFC analysis were positioned in the basolateral AY (Roy et al., 2009) and the body of HP (Kahn et al., 2008) (Figure 1). Both of these seeds are characterized by an extensive neocortical iFC pattern, which covers the above-mentioned intrinsic networks involved in MDD (Greicius et al., 2007; Sheline et al., 2010; Hamilton et al., 2011). Left and right basolateral AY were defined by the Anatomy Toolbox (Wellcome Department of Cognitive Neurology, London) in SPM and converted to ROIs using MarsBaR ^1^. The Anatomy Toolbox integrates probabilistic cytoarchitectonic maps derived from human post-mortem studies into the SPM environment. MarsBaR was used to create spherical ROIs (4 mm radius) with center coordinates (−24 −18 −18 and +24 −18 −18) in the body of the posterior HP (Kahn et al., 2008). After Butterworth bandpass-filtering of all voxel time courses for the frequency range from 0.009 to 0.08 Hz, we extracted voxel time courses of seed ROIs and reduced them to ROI-representative time courses by singular value decomposition, respectively. Each time course was put into a first-level fixed-effects general linear model in SPM8, and four separate iFC analyses (i.e., left AY, right AY, left HP, and right HP) were performed for each subject. For each model, additional regressors for global GM, white matter (WM), cerebral spinal fluid (CSF) BOLD-signal, and six movement parameters (three translational and three rotational directions) were included as covariates of no interest.

#### Group level iFC analysis

Group analyses were performed using contrast images from the subject-level iFC analysis in two separate flexible factorial models of ANOVA that included covariates of no interest [sex, age, and seed voxel-based morphometry (VBM) volume (see below for VBM analysis)]. The first ANOVA model was performed with factors group (with levels MDD group and healthy control group) and ROI (with levels left and right AY). The second ANOVA model was performed with factors group and ROI (with levels left and right HP). For both ANOVA models, appropriate *post hoc t*-tests were used to reveal the positively and negatively correlated iFC pattern for HP and AY that was representative of each study group (Roy et al., 2009). For both ANOVA models, the main effect of group (and corresponding *post hoc t*-tests to reveal direction of change) was the effect of interest. Statistical thresholds were set to *p* < 0.05 whole brain corrected for false discovery rate (FDR). Reported voxel coordinates correspond to standardized MNI space.

### Voxel-based morphometry

To determine structural HP and AY changes in MDD and to control for their effects on iFC results, VBM of structural MRI data was performed. VBM analysis followed a protocol described in previous work (Sorg et al., 2012). VBM8 toolbox ^2^ was used for data preprocessing and analysis. Images were corrected for bias-field inhomogeneity, registered using linear (12-parameter affine) and non-linear transformations, and tissue-classified into GM, WM, and CSF within the same generative model (Ashburner and Friston, 2005). GM images were modulated to account for volume changes based on the normalization process. We only considered non-linear volume changes so that subsequent analyses did not have to account for differences in head size. Finally images were smoothed with an 8-mm (FWHM) Gaussian kernel. Regional VBM volumes of HP and AY (mean of left and right HP and AY, respectively) were calculated for the body of HP and basolateral AY. Group differences were assessed by two-sample *t*-tests (*p* < 0.05). Regional VBM volumes were included as covariates-of-no interest in the group iFC analysis.

## Results

### Reduced HP and AY volume in patients

Regional volumes of both body of HP and basolateral AY were evaluated by comparing regional VBM volumes between patients with MDD and healthy subjects by the use of two-sample *t*-test. Both HP (*p* = 0.011) and AY (*p* = 0.019) volumes were reduced in patients (Figure 1).

### Overlapping reduced intrinsic connectivity of HP and AY in patients

**Figure 2:**
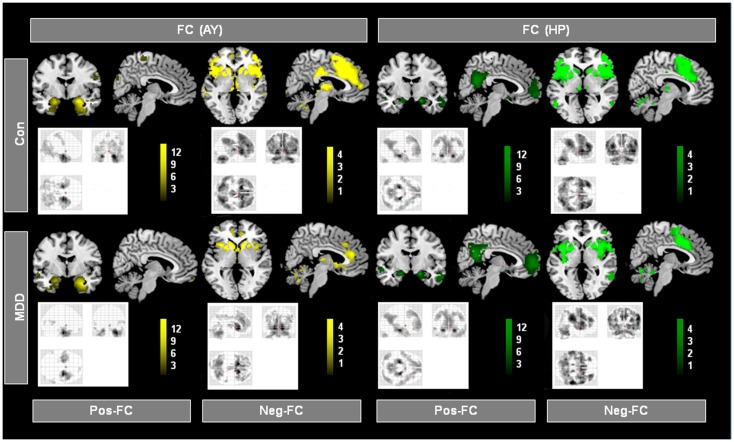
**Intrinsic functional connectivity (iFC) patterns of hippocampus (HP) and amygdala (AY) in patients and healthy controls**. Individual spatial ß-maps representing BOLD correlations of ongoing left and right HP and AY activity of patients with major depressive disorder (MDD) and healthy controls (Con), were analyzed using two ANOVA models with factors for group and hemisphere for the HP and AY, respectively. Yellow and green maps, which were superimposed on a single-subject high resolution T1 image, represent results of corresponding *post hoc t*-tests that reflect positive and negative FC (Pos-FC and Neg-FC, respectively) for each group and seed region (*p* < 0.05, FDR corrected; bars represent range of *t*-values).

**Figure 3:**
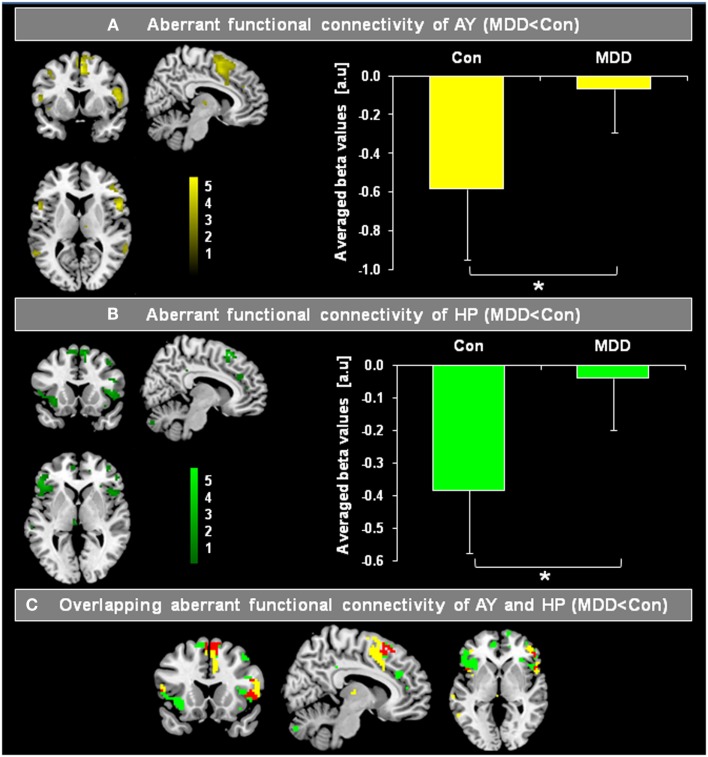
**Overlapping differences in the intrinsic functional connectivity (iFC) of the hippocampus (HP) and amygdala (AY) between patients with major depressive disorder (MDD) and healthy controls (Con)**. Individual spatial ß-maps, which represent BOLD correlations of ongoing left and right HP and AY BOLD activity of patients with major depressive disorder (MDD) and healthy controls (Con), were analyzed using two ANOVA models with factors for group and hemisphere for AY as well as HP. Yellow and green maps of **(A,B)** were superimposed on a single-subject’s high resolution T1 image, and represent the main effect of group on the FC of the AY and HP, respectively (*p* < 0.05, FDR corrected; bars represent range of *t*-values). Bar graphs on the right side reflect averaged iFC of AY and HP for MDD patients and healthy controls, respectively (two-sample *t*-tests, *p* < 0.05). In **(C)**, regions of overlapping reduced FC of AY **(A)** and HP **(B)** are shown in red. The HP and AY have reduced FC in the dorsomedial-prefrontal cortex and fronto-insular operculum in patients.

Figure 2 presents spatial maps of BOLD FC for the body of HP and basolateral AY in MDD patients and healthy controls. Maps are based on *post hoc t*-tests of different ANOVA models, which included corresponding covariates of regional seed VBM volumes (*p* < 0.05, FDR for multiple comparisons; Table 2). Results are largely consistent with previous findings (Kahn et al., 2008; Etkin et al., 2009; Roy et al., 2009; Cao et al., 2012). Ongoing AY activity was positively correlated with BOLD activity within the medial temporal lobe and in areas of primary sensorimotor and visual cortex in both patients and controls. HP activity was positively correlated with BOLD activity within regions of the default mode network such as the medial PFC and posterior cingulate cortex. Both AY and HP activity was negatively correlated with BOLD activity within regions of the salience network (such as dorsomedial PFC and FIO) and inferior parietal lobule. Differences between MDD and healthy participants were observed for HP and AY-FC within the dorsomedial PFC and FIO. Specifically, patients showed less negative BOLD-correlation between these brain regions and HP and AY. Further, these changes in HP and AY iFC overlapped (*p* < 0.05; FDR corrected; Figure 3, Table 2).

**Table 2:** **Regions of intrinsic functional connectivity (iFC) with amygdala (AY) and hippocampus (HP)**.

Anatomical region	L/R	Cluster	*T*-score	*p*-Value	Peak (MNI)
**A (I). POSITIVE IFC (AY), HEALTHY CONTROLS**					
Amygdala	R	649	10.27	<0.001	24, −3, −18
Parahippocampal cortex	R	649	6.18	<0.001	21, −18, −24
Superior temporal gyrus	R	649	7.20	<0.001	33, 6, −30
Amygdala	L	309	8.25	<0.001	−21, −3, −21
Parahippocampal cortex	L	309	8.09	<0.001	−24, −9, −18
Superior temporal gyrus	L	309	3.89	<0.001	−24, 9, −39
Medial frontal gyrus	R	11	3.77	0.008	3, −33, 69
Medial frontal gyrus	L	7	3.76	0.009	−3, −30, 66
Superior occipital gyrus	R	157	4.85	<0.001	42, −63, 0
Cuneus	L	10	4.10	0.003	−27, −87, 33
Cuneus	R	56	3.95	0.005	27, −84, 27
**A (II). NEGATIVE IFC (AY), HEALTHY CONTROLS**					
Fronto-insular operculum	L	1701	6.33	<0.001	−45, 15, 0
Inferior frontal gyrus	L	1701	6.05	<0.001	−45, 12, 45
Putamen	L	1701	8.97	<0.001	−18, 15, −3
Fronto-insular operculum	R	3526	7.77	<0.001	54, 15, 6
Inferior frontal gyrus	R	3526	6.42	<0.001	45, 39, 0
Medial frontal gyrus	L/R	3526	7.92	<0.001	0, 39, 24
Putamen	R	3526	8.36	<0.001	15, 18, 0
Cingulate cortex	R/L	3526	6.30	<0.001	3, 24, 36
Thalamus	R/L	179	7.16	<0.001	9, −15, 6
Inferior parietal lobule	R	529	6.56	<0.001	54, −36, 39
Angular gyrus	L	417	5.70	<0.001	−57, −48, 33
Posterior cingulate cortex	L/R	161	5.15	<0.001	0, −30, 27
**A (III). POSITIVE IFC (HP), HEALTHY CONTROLS**					
Parahippocampal cortex, HP	L	1404	12.85	<0.001	−21, −18, −18
Parahippocampal cortex, HP	R	1404	12.22	<0.001	24, −18, −18
Posterior cingulate cortex	L/R	1404	7.71	<0.001	6, −54, 18
Angular gyrus	R	131	6.33	<0.001	48, −63, 27
Superior frontal gyrus	R	87	6.18	<0.001	24, 30, 45
Medial frontal gyrus	L/R	170	5.76	<0.001	0, 60, −6
Angular gyrus	L	172	5.73	<0.001	−39, −72, 33
Middle temporal gyrus	R	111	5.64	<0.001	63, −6, −27
**A (IV). NEGATIVE IFC (HP), HEALTHY CONTROLS**					
Fronto-insular operculum	R	5639	9.43	<0.001	45, 18, 6
Middle frontal gyrus	R	5639	7.63	<0.001	45, 48, 9
Inferior frontal gyrus	R	5639	7.69	<0.001	51, 39, 3
Inferior parietal lobule	R	5639	7.86	<0.001	60, −36, 33
Medial frontal gyrus	R	5639	6.77	<0.001	3, 9, 63
Anterior cingulate cortex	R	5639	4.55	<0.001	9, 33, 27
Fronto-insular operculum	L	2342	7.73	<0.001	−36, 15, 0
Dorsolateral prefrontal cortex	L	2342	9.18	<0.001	−36, 42, 24
Middle frontal gyrus	L	2342	7.69	<0.001	−35, 38, 30
Inferior frontal gyrus	L	2342	6.51	<0.001	−48, 33, 0
Medial frontal gyrus	L	2342	5.77	<0.001	−3, 3, 60
Inferior parietal lobule	L	2342	6.92	<0.001	−57, −39, 33
Middle temporal gyrus	L	127	4.92	<0.001	−57, −57, 0
**B (I). IFC (AY): MDD < HEALTHY CONTROLS**					
Medial frontal gyrus	R	27	5.09	0.020	3, 9, 51
Superior frontal gyrus	R	27	3.81	0.020	9, 27, 54
Fronto-insular operculum	R	36	4.97	0.020	54, 15, 6
Inferior frontal gyrus	R	9	4.46	0.030	48, 36, 3
Inferior parietal lobule	L	4	4.40	0.030	−60, −51, 33
**B (II). IFC (HP): MDD < HEALTHY CONTROLS**					
Superior frontal gyrus	R	20	5.17	0.017	9, 21, 60
Fronto-insular operculum	R	10	4.12	0.028	45, 18, 6
Inferior frontal gyrus	L	66	4.90	0.022	−51, 33, 0
Medial frontal gyrus	L	23	4.72	0.025	−9, 24, 60
Middle frontal gyrus	L	21	4.45	0.026	−39, 9, 42
Middle frontal gyrus	L	47	4.38	0.026	−45, 36, 27
Inferior parietal lobule	R	28	4.33	0.026	51, −45, 36
**B (III). IFC (OVERLAP): MDD < HEALTHY CONTROLS**					
Superior frontal gyrus	R				9, 21, 54
Medial frontal gyrus	R				9, 21, 51
Medial frontal gyrus	L				−3, 24, 54
Inferior frontal gyrus	R				51, 15, 3
Fronto-insular operculum	R				42, 21, 3

Healthy controls and group differences with MDD patients.

A: one-sample *t*-tests, *p* < 0.05, corrected for false discovery rate (FDR). B: two-sample *t*-tests, *p* < 0.05, FDR corrected; MDD, recurrent major depressive disorder; iFC, intrinsic functional connectivity; AY, amygdala; HP, hippocampus; L, left; R, right.

## Discussion

The present study investigated differences in functional connectivity of HP and AY in patients with MDD and healthy controls to provide new insight into the pathophysiology of MDD. We found that ongoing BOLD activity within the body of HP and basolateral AY was negatively correlated with activity within the dorsomedial PFC and FIO. However, the negative FC between these brain regions was weaker in MDD, independent of patients’ reduced HP and AY GM volumes. These findings provide the first evidence that the HP and AY show a corresponding reduction in iFC to other brain regions in MDD. Since dorsomedial PFC and FIO are critical for intrinsic network interactions (i.e., default mode, salience, and central executive network), which are impaired in MDD, these findings suggest that aberrant HP and AY intrinsic connectivity may interact with aberrant intrinsic network activity in MDD.

### Overlapping aberrant HP and AY intrinsic connectivity to prefrontal cortex and fronto-insular operculum in MDD

The iFC analyses of the present study revealed that during the resting-state, both HP and AY BOLD-functional connectivity with dorsomedial PFC and FIO was reduced in MDD (Figure 3; Table 2). Healthy controls showed a strong negative correlation between basolateral AY and the dorsolateral PFC, dorsomedial PFC, FIO, and inferior parietal lobule (Figure 2). However, AY-FC with the dorsomedial PFC, FIO, and inferior frontal gyrus were substantially reduced in patients. A very similar, and importantly, overlapping pattern was observed in the iFC analysis for the HP (Figure 3; Table 2). While healthy control participants showed a strong negative correlation between HP and the PFC (i.e., dorsomedial PFC and FIO) (Figure 2), patients showed weaker FC between the HP and these brain regions. Reduced FC of both AY and HP to PFC and FIO cannot be explained by decreased AY and HP volumes in patients, as these volumes were included in the iFC analyses as covariates of no interest. Each of these brain regions has been implicated in emotion-related processes (Ochsner and Gross, 2005; Wood et al., 2012). Further, these brain regions appear to play a role in the regulation of emotion (Ochsner and Gross, 2005). For example, both medial and lateral regions of the PFC support processes related to the cognitive control of emotion (Ochsner and Gross, 2005; Kalisch, 2009; Winecoff et al., 2011). Furthermore, AY-FC during emotion regulation was decreased within the FIO and dorsomedial PFC in young MDD patients, perfectly matching our finding (Perlman et al., 2012).

Similarly, it has been demonstrated that there is a robust decrease of resting-state functional connectivity of bilateral amygdala, anterior insula, and anterior cingulate cortex within the salience network and ventromedial prefrontal cortex (vmPFC) and temporal poles in medication-free MDD patients with no comorbidity (Veer et al., 2010). In addition, Cao et al. (2012) found that drug-naïve patients with MDD showed selectively less negative BOLD-correlation between ongoing HP and lateral PFC activity, while Tang et al. (2012) observed reduced FC between AY and ventral PFC. The findings from both of these prior studies are consistent with our current results. In summary, our findings demonstrate there is significant overlap in the aberrant FC of the HP and AY to both the dorsomedial PFC and FIO in MDD.

A key finding in the present study is the reduction in both HP and AY iFC with the dorsomedial PFC in MDD patients. The HP and AY have well established roles in learning, memory, and particularly emotional memory (LaBar and Cabeza, 2006; LeDoux, 2007). Impaired emotional memory may play a critical role in the pathogenesis of MDD (Segal et al., 1996; Kendler et al., 2001; Robinson and Sahakian, 2008). Further, recent research indicates that depression is associated with aberrant iFC in the PFC (referred to as the dorsal nexus) within several intrinsic networks (Sheline et al., 2010). More specifically, depression is associated with an increase in iFC between the dorsomedial PFC and default mode, salience, and executive control networks (Sheline et al., 2010; Scheidegger et al., 2012). This prior work suggests that aberrant functional connectivity between the dorsomedial PFC and these intrinsic brain networks may be an important aspect of the pathophysiology of depression. These changes in functional connectivity may provide a mechanism that explains the co-occurrence of depressive symptoms, such as increased self-focus, that are mediated by these distinct brain networks (Sheline et al., 2010). The present study extends this prior work by demonstrating that both the HP and AY also show aberrant iFC with the dorsomedial PFC in MDD patients. The changes observed in patients’ HP and AY iFC with the dorsomedial PFC (i.e., the dorsal nexus) may provide a mechanism by which disruptions in emotional memory act synergistically with other cognitive and behavioral symptoms of depression.

The HP and AY also showed aberrant iFC with the FIO in patients compared to healthy participants. More specifically, both the HP and AY showed reduced iFC with the FIO. The FIO is considered the hub of a neural circuit that detects salient events (i.e., the salience network), facilitates access to attention and working memory resources, and interacts with other intrinsic networks (i.e., the default mode and central executive network) to generate appropriate responses to salient stimuli (Seeley et al., 2007; Menon, 2011). Psychopathological processes may be mediated by dysfunction of the salience network. Given that a minimal amount of salience network activity is necessary for healthy cognitive-emotional functioning, a decrease in network activity may result in psychopathology (e.g., depression). For example, prior work suggests the FIO initiates adaptive emotion regulation and network interaction processes that are triggered by the rumination of depressed individuals (Hamilton et al., 2011). In the present study, the HP and AY showed impaired iFC to the FIO in MDD patients. This reduction in iFC to the salience network may provide a mechanism for the impaired emotional memory associated with the HP and AY dysfunction that contributes to impaired salience detection. Thus, disruption of salience detection processes via decreased HP and AY iFC to the FIO may play a critical role in the pathophysiology of MDD.

Voxel-based morphometry findings from the current study replicate prior research investigating HP and AY volume changes associated with MDD, indicating the representative character of our patient sample and thereby of our iFC results. We observed AY atrophy in MDD patients compared to controls (Figure 1). Findings from prior research investigating changes in AY volume associated with MDD have been mixed (Frodl et al., 2003, 2008a,b; Kempton et al., 2011). In addition, we observed significant HP atrophy in MDD patients compared to healthy controls (Figure 1). Similar results have been demonstrated in prior MDD neuroimaging studies (Sheline et al., 1996; Kronmuller et al., 2008). These findings provide further evidence that structural changes in the HP are associated with MDD.

### Limitations and methodological issues

Patients in our study were treated with antidepressant medications. It is difficult to statistically control for antidepressant effects because there is no reliable tool available to make drug effects comparable across different antidepressants [in contrast to antipsychotic drugs, which can be mapped on chlorpromazine equivalents CPZ and included as additional covariates in analyses (e.g., Sorg et al., 2012)]. Due to the complexity of this issue, the impact of antidepressants on iFC is not completely understood (Bruhl et al., 2010; Delaveau et al., 2011). Recent studies suggest antidepressant medications normalize brain function (Anand et al., 2005; Fu et al., 2007; Heller et al., 2013). Consistent with this suggested normalization of function, the reduction in HP and AY iFC in medicated patients in the present study is largely consistent with previous findings in treatment-naïve patients with MDD (Cao et al., 2012; Tang et al., 2012). For example Cao et al. (2012) reported weaker negative BOLD-correlations between the HP and the middle frontal gyrus in non-medicated patients that are similar to our present findings in medicated patients. However, although our findings fit previous results, we cannot exclude medication effects for our results. Therefore, the present results should be interpreted with caution. Future studies in non-medicated patients are necessary to replicate and extend our findings.

We used standard rs-fMRI analysis procedures to calculate our main outcome measure of ROI-based FC ß-maps. These procedures included global signal regression to remove physiological noise (such as respiratory and cardiac based signals) from the resting-state signal. However, there is an ongoing debate regarding whether global signal regression induces artificial BOLD correlations, particularly negative correlations (Murphy et al., 2007; Chang and Glover, 2009; Fox et al., 2009; Chai et al., 2012). We decided to use global signal regression for the following reasons: (i) recent studies have demonstrated the biological origin of negatively correlated BOLD FC during rest that is independent of global signal regression application (Chai et al., 2012; Wong et al., 2012). (ii) Particularly for the AY, negative BOLD correlation with frontolimbic areas have recently been found in awake rats, independent of global signal regression (Liang et al., 2012). This finding demonstrates the robustness of the negative BOLD-correlation network seeded by AY across species, as well as its independence from global signal regression. (iii) Previous studies in healthy controls and MDD used global signal regression when investigating the iFC of the medial temporal lobe (Roy et al., 2009; Cao et al., 2012). Therefore, to better compare our findings with these prior studies, we used the same approach.

## Conclusion

In patients with MDD, reduced HY and AY intrinsic connectivity overlaps within the dorsomedial PFC and FIO. These regions not only support the expression and regulation of emotion, which is impaired in MDD, but also regulate intrinsic brain network interactions. Since these networks and their interactions are disrupted in MDD, our finding suggests that there is a link between the medial temporal lobe and intrinsic network pathophysiology in MDD that is mediated aberrant HP and AY intrinsic connectivity.

## Conflict of Interest Statement

The authors declare that the research was conducted in the absence of any commercial or financial relationships that could be construed as a potential conflict of interest.

## Footnotes

^1^http://marsbar.sourceforge.net

^2^http://dbm.neuro.uni-jena.de/vbm.html
